# Bats Avoid Radar Installations: Could Electromagnetic Fields Deter Bats from Colliding with Wind Turbines?

**DOI:** 10.1371/journal.pone.0000297

**Published:** 2007-03-14

**Authors:** Barry Nicholls, Paul A. Racey

**Affiliations:** School of Biological Sciences, University of Aberdeen, Aberdeen, United Kingdom; University of St. Andrews, United Kingdom

## Abstract

Large numbers of bats are killed by collisions with wind turbines, and there is at present no direct method of reducing or preventing this mortality. We therefore determine whether the electromagnetic radiation associated with radar installations can elicit an aversive behavioural response in foraging bats. Four civil air traffic control (ATC) radar stations, three military ATC radars and three weather radars were selected, each surrounded by heterogeneous habitat. Three sampling points matched for habitat type and structure, dominant vegetation species, altitude and surrounding land class were located at increasing distances from each station. A portable electromagnetic field meter measured the field strength of the radar at three distances from the source: in close proximity (<200 m) with a high electromagnetic field (EMF) strength >2 volts/metre, an intermediate point within line of sight of the radar (200–400 m) and with an EMF strength <2 v/m, and a control site out of sight of the radar (>400 m) and registering an EMF of zero v/m. At each radar station bat activity was recorded three times with three independent sampling points monitored on each occasion, resulting in a total of 90 samples, 30 of which were obtained within each field strength category. At these sampling points, bat activity was recorded using an automatic bat recording station, operated from sunset to sunrise. Bat activity was significantly reduced in habitats exposed to an EMF strength of greater than 2 v/m when compared to matched sites registering EMF levels of zero. The reduction in bat activity was not significantly different at lower levels of EMF strength within 400 m of the radar. We predict that the reduction in bat activity within habitats exposed to electromagnetic radiation may be a result of thermal induction and an increased risk of hyperthermia.

## Introduction

Bats are substantially more vulnerable to collisions with wind turbines than birds, although the underlying reasons for collision mortalities remain unclear [1], [2]. The scale of the problem became apparent recently when, during a six-week period, an estimated 1,764 and 2,900 bat fatalities were recorded at two wind farms in West Virginia and Pennsylvania [2]. Bat fatality was highest during late summer and fall when bats begin autumn migration [3] and migratory species comprise the majority of fatalities at all wind farms studied to date [4], [5], [6]. This is undoubtedly exacerbated by the placement of wind turbines on topographical features such as ridgelines and in forest corridors, which are used as migratory routes for several bat species [7], [8]. However, it remains unclear whether foraging bats, as well as migrating individuals, are also at risk from collisions. Thermal images of wind turbines appear to indicate that bats are attracted to and investigate both moving and static blades [2], and studies in Europe have also reported bats foraging close to turbine blades [9], [10], [11].

The numbers of collision mortalities reported in America are much greater than in Europe, where mortality surveys have begun more recently. One of the problems with providing accurate data on bat deaths in Europe is the lack of a consistent methodology. Numbers in published reports range from 2–50 bats with each study including a different number of turbines, different survey methods and different time periods [10], [12], [13]. However, 15 of the 35 species of European bat have been recorded as regular victims of turbine collisions, and an Intersessional Working Group of Eurobats cited 20 species thought to be at risk of collision [14].

Current research in Europe is concentrated on the development of scientifically credible mortality estimates to assess the extent of the problem. Although this is clearly important, the rapid proliferation of wind turbines requires a more urgent response. Research is required on the underlying reasons behind these collisions and on potential methods to mitigate this increasing threat to endangered bat populations.

Attempts at mitigating bird collisions with wind turbines have typically involved the application of visual stimuli to increase the conspicuousness of the turbine blades [15], [16], but for bats, where audition is the primary sensory modality, this is clearly inappropriate. The design of an acoustic deterrent for bats, as used to mitigate cetacean entanglement in drift nets [17], [18], [19], is complicated by the intrinsic properties of ultrasound, which attenuates rapidly in air [20]. Therefore in the absence of an efficient acoustic deterrent it is essential to investigate alternative sources capable of inducing aversive behaviour in bats.

Researchers at Aberdeen University have observed for some time that bat activity is reduced in the vicinity of the Aberdeen civil Air Traffic Control (ATC) radar station despite the proximity of habitat types where bat activity would be expected. This raised the possibility that the radio frequency (RF) radiation associated with radar installations may elicit an aversive behavioural response in foraging bats. Radio frequencies occupy the portion of the electromagnetic spectrum between 3 kHz and 300 GHz. Absorption of energy in the range of 1 MHz–300 GHz results primarily in tissue heating by movement of ions and oscillations of dipole molecules resulting in a transfer of energy from the RF field to the biological medium [21]. Short term RF exposure can produce a thermal burden in an organism that can result in significant behavioural and physiological changes, some of which may be harmful [22]. Although behavioural effects of such exposure on humans include perception, aversion, work perturbation, work stoppage and convulsions [23], few field experiments have been carried out to ascertain the possible effects of high frequency electromagnetic radiation on wild animals. However, electromagnetic radiation can influence the development, reproduction, and physiology of insects [24], mammals [25], and birds [26]. There is no current direct evidence to suggest that bats can detect or respond to electromagnetic radiation. However, we predict that if high frequency electromagnetic radiation exerts an aversive response in foraging bats, bat activity will be reduced in the vicinity of radar installations. The aim of the present study was to test this hypothesis.

## Methods

### Study sites and sampling protocol

**Figure 1:**
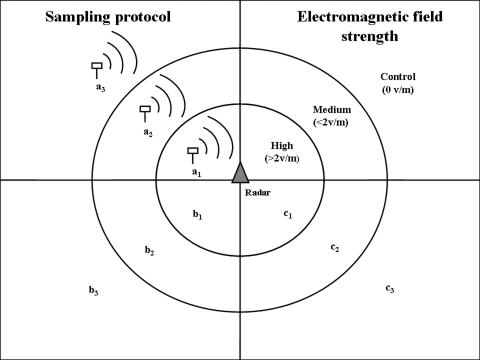
Sampling protocol of surveys carried out at ten radar stations from June to September 2006. At each radar station bat activity was recorded at three matched sites along an electromagnetic gradient (a_1_,a_2_,a_3_). Each radar installation was surveyed on three occasions throughout the study with three different sampling points (a,b,c) sampled on each occasion.

In Britain, foraging bats are predominantly associated with areas where insect density is high: broadleaved woodland, particularly woodland edge, linear vegetation (tree lines and hedgerows) and riparian habitat [27]. More open and intensively managed areas are avoided [28]. Therefore in order to assess the impact of radar on foraging bats it was important to locate radar stations surrounded by habitat suitable for foraging. This negates the possibility that the absence of bats around radar stations is simply an artefact of the exposed location of the radar. Following initial reconnoitres, 10 radar stations were selected (Table 1). These included four civil airport air traffic control (ATC) radar stations, three military ATC radars and three weather radars. Each selected radar station was surrounded by habitat with a high degree of heterogeneity, thereby facilitating the identification of sampling points along an electromagnetic gradient. At each radar station three sampling points were chosen within one of three habitat categories: riparian woodland, woodland edge and tree-lines. Each sampling point was matched for habitat type, habitat structure (e.g. height and length of isolated tree-line), dominant vegetation species, altitude and surrounding land class. Each successive sampling point was located at increasing distance from the radar station and subject to differing levels of electromagnetic field strength (Fig. 1).

**Table 1:** Location, category and operating frequency of the ten radar installations.

Sampling Site	Radar Station	Radar Type	Radar Band (operating frequency)	Grid reference
1	Perwinnes hill	civil ATC	L band (1–2 GHz)	NJ 922 132l
2	Allenshill	civil ATC	L band (1–2 GHz)	NJ 905 602
3	Prestwick	civil ATC	S band (2–4 GHz)	NS 367 279
4	Lowther hill	civil ATC	L band (1–2 GHz)	NS 888 106
5	RAF Leuchars	military ATC	S band (2–4 GHz)	NO 470 208
6	RAF Lossiemouth	military ATC	S band (2–4 GHz)	NJ 207 694
7	RAF Buchan	military ATC	S band (2–4 GHz)	NK 113 408
8	Balado bridge	weather radar	S band (2–4 GHz)	NO 094 028
9	Hill of Dudwick	weather radar	S band (2–4 GHz)	NJ 979 377
10	Corse hill	weather radar	S band (2–4 GHz)	NS 594 463

A portable electromagnetic field meter (PMM 8053-Accelonix Ltd.) and isotropic field probe (EP-330 Isotropic E-Field probe-Accelonix Ltd.) were used to measure the electromagnetic field strength (EMF) of the radar in volts per metre (v/m) at three distances from the radar source. Radars emit a train of very brief pulses of high intensity followed by a silent interval for the echoes to return, therefore the peak intensity, at each sampling point, was recorded in one minute intervals over a thirty minute period and the average of these readings used to classify each site as follows: in close proximity to the radar (<200 m) and subject to a high electromagnetic field (EMF) strength >2 v/m, an intermediate sampling point within line of sight of the radar (200–400 m) and with an EMF strength <2 v/m and a control site that was not in line of sight of the radar and registered an EMF of zero v/m (>400 m).

At each radar station bat activity was recorded contemporaneously within these three field strength categories. Paired sampling was used to control for variation in bat activity due to environmental parameters. Throughout the summer each radar station was surveyed on three occasions with three different sampling points monitored on each occasion (Fig. 1) resulting in a total of 90 samples, 30 of which were obtained within each field strength category.

### Bat activity recording

At each radar station bat activity was recorded using three automatic bat-recording stations [29]. Each automatic station consisted of a Batbox 3 heterodyne bat detector (Stag Electronics, Sussex, UK) linked to a count data logger (Gemini Data Loggers, UK Ltd, Chichester, UK) via an analogue to digital signal converter (Skye instruments, Ltd). The signal converter converts analogue signals from the bat detector into digital signals that can be recorded by the data logger. Every 0.5 seconds a positive or negative signal is sent to the data logger indicating the presence or absence of ultrasound respectively. The Batbox 3 was tuned to 50 kHz in order to detect each of the five breeding species of bat in Scotland (*Pipistrellus pipistrellus, Pipistrellus pygmaeus, Myotis daubentonii, Myotis nattereri* and *Plecotus auritus*). However *P. auritus* seldom emits calls loud enough to be detected and is therefore unlikely to be recorded. The recording stations were operational from sunset to sunrise and the data loggers were set to record bat active minutes (the number of minutes throughout the recording period that ultrasound was detected by the bat detector). The component parts of the system were housed in large plastic boxes with a hole cut for the bat detector microphones. Automatic recording stations were positioned on platforms 1.5 m above the ground and orientated perpendicular to the linear element (e.g. woodland edge, tree-lines, riparian woodland).

In addition to the automatic recording of bat activity, a 30 minute transect was carried out at each sampling site using a bat detector (S-25 Ultrasound Advice, London) set to frequency division mode. This method of ultrasound transformation allows calls to be recorded in real time on audiocassettes and the number of recorded passes provides a quantitative assessment of bat activity [30]. Bat detectors were linked to a tape recorder (Sony Walkman, Tokyo, WMD6C) containing metal cassettes. At each site, sound recording equipment was held at waist height and a 50 m transect was walked in a zigzag fashion back and forth across the site to avoid any bias in direction or placement of the detectors. The 30 minutes recording at each site was analysed using BatSound Pro software (Pettersson Elektronic AB, Uppsala Sweden).

### Statistical analysis

Bat activity in sites subject to electromagnetic radiation (>2 v/m) was compared to the control sites (0 v/m) using paired t tests. To avoid pseudoreplication, tests were carried out on the average of the three replicates at each radar site (n = 10). Data were log (log_10_ (x+1)) transformed to achieve normality and homogeneity of variance [31]. Analyses were carried out using Minitab version 14 [32]. The difference in bat activity between treatment and control groups was analysed further using general linear models (GLMs) in SPSS 12.0, including all relevant and recorded confounding variables. Radar type was included as a random factor and reproductive status (pre-lactation, lactation, post-lactation), temperature and EMF strength (high or intermediate) were included as covariates. Interactions were only included in the model when of direct relevance to the hypothesis being tested and interactions between confounding variables were not tested.

## Results

The automatic stations recorded a total of 3727 bat active minutes over 230 h of recording. A further 2979 bat passes were recorded during transects with the frequency division detector (45 h). As expected, the majority of passes (83%) were attributed to the two cryptic pipistrelle species: *Pipistrellus pipistrellus* and *Pipistrellus pygmaeus* (45% and 38% respectively) which are the most common and abundant bats in Scotland. A further 14% of bat passes were attributed to *Myotis daubentonii* and 3% to *Myotis nattereri*.

### Bat activity

**Figure 2:**
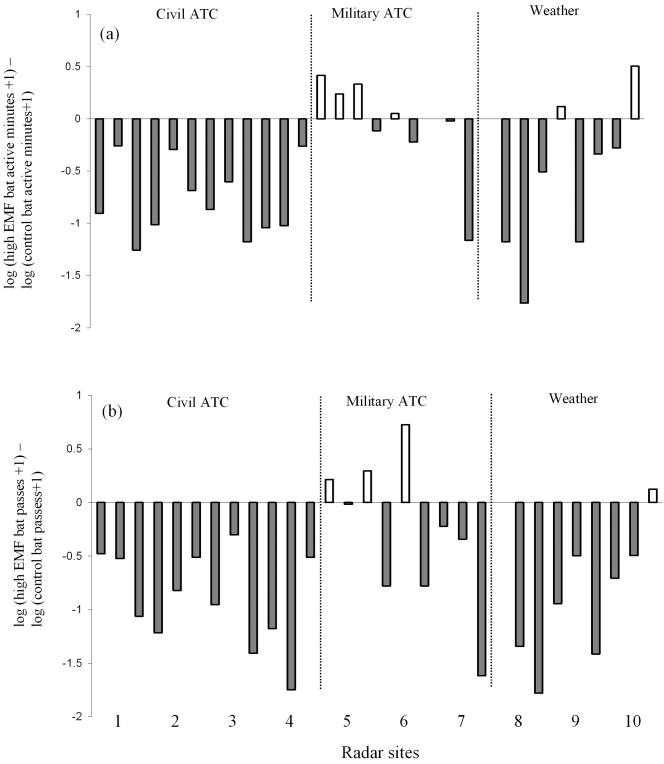
The differences in: (a) log bat active minutes (high EMF minus control). A negative value indicates that bat activity was higher at the control site than at the site subject to a high electromagnetic field strength (>2 v/m). (b) log total number of bat passes (high EMF minus control). Each triad of differences represent a single radar site (n = 10).

Total bat activity (bat active minutes) was higher in the control sites (0 v/m) when compared to sites exposed to a high level (>2 v/m) of electromagnetic radiation. Paired t tests carried out on the log-transformed bat active minutes, recorded by the automatic bat recording stations, showed that bats were significantly more active in control sites when compared to high EMF sites (t = 4.41; n = 10; p = 0.003; Fig. 2a). The number of bat passes (all species) recorded during transects with the frequency division detector was also higher in the control sites when compared to sites exposed to a high level of electromagnetic radiation. Paired t tests carried out on the log transformed number of bat passes, showed that bats were significantly more active in control sites when compared to high EMF sites (t = 4.86; n = 10; p = 0.001; Fig. 2b).

The general linear models showed that, when confounding variables were taken into account, the level of EMF strength within 400 m of the radar had no significant effect on the difference in bat activity recorded between treatment and control groups (Table 2). However, radar type did have a significant effect (Table 2) with the difference in bat activity between treatment and control groups greatest in the vicinity of civil ATC radars and least in the vicinity of military ATC radars (Fig 2). The interaction of field strength*radar type was not significant when added to the model (Bat active minutes: F_2,50_ = 2.3, p = 0.11; bat passes: F_2,50_ = 0.3, p = 0.76) indicating that each radar type had a similar effect at both high and intermediate EMF sites.

**Table 2:** General linear models for the difference in bat activity between treatment (high or intermediate EMF sites) and control groups, investigating the effect of EMF strength.

	Source of variation	Sum of squares (type3)	***F***	d.f.	***P***
**Bat active minutes**					
	Intercept	6 436	4.7	1	0.036
	Intercept error	56 577		50	
Fixed:	Habitat	3 136	1.2	2	0.293
Random:	Radar type	9 278	3.7	2	0.031
Covariates:	EMF strength	260	0.2	1	0.65
	Temperature	21 137	16.9	1	>0.001
	Repro. Status	300	0.24	1	0.625
**Bat passes**					
	Intercept	13 295	5.115	1	0.028
	Intercept error	120 235		46.2	
Fixed:	Habitat	10 654	2.2	2	0.115
Random:	Radar type	22 879	4.8	2	0.012
Covariates:	EMF strength	277	0.1	1	0.733
	Temperature	28 574	12.0	1	0.001
	Repro. Status	5 875	2.5	1	0.121

## Discussion

### Bat activity and foraging effort

Currently there have been no successful attempts to directly mitigate bat collisions with wind turbines. Attempts at deterring bats by the use of ultrasound have, as yet, been unsuccessful. Therefore the identification of alternative methods capable of inducing an aversive response in bats approaching turbine blades is of paramount importance. Our result have demonstrated that bat activity is significantly reduced in habitats exposed to an electromagnetic field (EMF) strength of greater than 2 v/m when compared to matched sites registering EMF levels of zero. Even at sites with lower levels of EMF exposure (<2 v/m), bat activity was reduced in comparison to control sites.

However, the difference in bat activity between treatment groups and control groups varied between radar types with results more equivocal in the vicinity of military ATC radars. It is possible that this may be explained by the characteristics and operating times of the individual radar units concerned, sensitive information which was not available. Despite this the overall reduction in bat activity within habitats exposed to high and intermediate EMFs supports our hypothesis that the electromagnetic radiation associated with radar installations can exert an aversive behavioural response in bats. This raises questions regarding the mechanisms by which bats could perceive electromagnetic radiation and why they would avoid EMF exposure. We propose two mechanisms through which electromagnetic fields could induce an aversive behavioural response in foraging bats: thermal induction leading to an increased risk of overheating and hyperthermia, and echolocation interference - the auditory microwave hypothesis.

### Thermal effects of EMF exposure

Studies investigating the behavioural response of laboratory animals to the presence of electromagnetic fields have provided substantial insight into the most probable mechanism of interaction of these fields with intact organisms. This mechanism relates to the generation of heat in the skin that results in the activation of thermal sensors in the tissues and central nervous system. Studies of human thermal sensation generated by RF exposures [33], [34], [35] reinforce the conclusion that behavioural changes observed in RF-exposed animals are thermally motivated. Indeed, it has been demonstrated in the laboratory that measured elevations of surface and deep body temperatures often accompany specific behavioural changes [36]. The effect advances from the threshold of perception, through intermediate steps, to an extreme thermal insult, grand mal seizures, and finally death. In this respect, exposure to an RF field differs little from exposure to conventional sources of thermal energy or inhospitable thermal environments [21].

For the majority of animals a short period of overheating constitutes a much greater hazard than does an equivalent degree of cooling [37], [38]. A rise of only a few degrees above the optimum temperature is quickly fatal. The wing membranes of bats present a large surface area over which radiation might be absorbed, increasing heat load to the animal. This, combined with the heat energy produced during flight makes bats particularly susceptible to overheating [39], [40], [41], which can be fatal in experimental conditions between 38–39°C [42]. Furthermore, observations of captive bats have noted their aversion to even a moderate infra-red heat source [37]. Therefore it is possible that thermal induction, resulting from EMF exposure in the vicinity of radar installations, may provide an inhospitable thermal regime for foraging bats, which could vary from discomfort to hyperthermia depending on EMF strength and the duration of exposure.

### Auditory microwave hypothesis

Although the mammalian ear has no sensitivity to electromagnetic waves at microwave frequencies (300 MHz–300 GHz) human auditory perception of radio frequency energy has been reported since the 1940s [43]–[48]. It is now widely accepted that the auditory perception of microwaves is a result of thermoelastic expansion [49], [50]. The absorption of the energy in the RF pulse leads to a rapid thermal expansion resulting in a thermoelastic wave. This wave is then propagated through the soft tissues of the head until it reaches the fluid-filled inner ear, where it is transduced into a sound pressure wave leading to excitation of auditory neurons in the kHz range [46], [51], [48]. Laboratory experiments have shown that the frequency of the induced sound is a function of head size and of the acoustic properties of the brain tissue. The estimated fundamental frequency of vibration in guinea pigs, cats and adult humans were 45, 38, and 13 kHz respectively [52], [53]. It is therefore not only plausible but probable that bats exposed to an RF pulse of sufficient power would effectively hear this pulse and the frequency detected would lie within the range of frequencies used for orientation, prey detection and capture for the majority of bat species. There is no evidence that the auditory perception of microwaves would act to deter foraging bats any more than the production of ultrasound at the same frequency. However if bats can perceive areas of high EMF exposure and experience an associated rise in internal temperature it provides a mechanism through which an aversive response may be elicited.

### Conclusions

We have demonstrated that bat activity is reduced in habitats exposed to electromagnetic radiation when compared with matched sites where no such radiation can be detected. However without access to detailed specifications of individual radar units (including operational times and operating frequency) it is difficult to quantify this relationship further. To more fully define the impact of radar on foraging bats, and ascertain its value as a potential source of mitigation, field trials involving a mobile radar that can be introduced into areas of known bat activity are now required. If the parameters of an RF signal capable of inducing an aversive response in foraging bats could be characterised then this may offer a method of mitigating bat collisions with wind turbines.
